# TIMER: A Clinical Study of Energy Restriction in Women with Gestational Diabetes Mellitus

**DOI:** 10.3390/nu13072457

**Published:** 2021-07-18

**Authors:** Efrosini Tsirou, Maria G. Grammatikopoulou, Meletios P. Nigdelis, Eleftheria Taousani, Dimitra Savvaki, Efstratios Assimakopoulos, Apostolos Tsapas, Dimitrios G. Goulis

**Affiliations:** 1 Unit of Reproductive Endocrinology, 1st Department of Obstetrics and Gynecology, Medical School, Faculty of Health Sciences, Aristotle University of Thessaloniki, GR-56429 Thessaloniki, Greece; efro_tsi@yahoo.gr (E.T.); mariagram@auth.gr (M.G.G.); meletis.nigdelis@gmail.com (M.P.N.); liataou@yahoo.gr (E.T.); dsavvaki@phyed.duth.gr (D.S.); 2 Department of Nutritional Sciences & Dietetics, Faculty of Health Sciences, Alexander Campus, International Hellenic University, GR-57400 Thessaloniki, Greece; 3 Department of Midwifery, Faculty of Health Sciences, Alexander Campus, International Hellenic University, GR-57400 Thessaloniki, Greece; 4 School of Physical Education and Sports Science, Democritus University of Thrace, GR-69100 Komotini, Greece; 5 2nd Department of Obstetrics and Gynecology, Hippokratio General Hospital, Aristotle University of Thessaloniki, 49 Konstantinoupoleos Str, GR-54642 Thessaloniki, Greece; assimakopoulose@gmail.com; 6 Clinical Research and Evidence-Based Medicine Unit, Hippokration Hospital, Aristotle University of Thessaloniki, 49 Konstantinoupoleos Str, GR-54642 Thessaloniki, Greece; atsapas@auth.gr; 7 Harris Manchester College, University of Oxford, Oxford OX1 3TD, UK

**Keywords:** pregnancy, physical activity, lifestyle, diet, caloric intake, low-calorie diet, diabetes mellitus, gestational weight gain, depression, obstetrical outcome

## Abstract

Medical nutrition therapy is an integral part of gestational diabetes mellitus (GDM) management; however, the prescription of optimal energy intake is often a difficult task due to the limited available evidence. The present pilot, feasibility, parallel, open-label and non-randomized study aimed to evaluate the effect of a very low energy diet (VLED, 1600 kcal/day), or a low energy diet (LED, 1800 kcal/day), with or without personalized exercise sessions, among women with GDM in singleton pregnancies. A total of 43 women were allocated to one of four interventions at GDM diagnosis: (1) VLED (*n* = 15), (2) VLED + exercise (*n* = 4), (3) LED (*n* = 16) or (4) LED + exercise (*n* = 8). Primary outcomes were gestational weight gain (GWG), infant birth weight, complications at delivery and a composite outcomes score. Secondary outcomes included type of delivery, prematurity, small- for-gestational-age (SGA) or large-for-gestational-age (LGA) infants, macrosomia, Apgar score, insulin use, depression, respiratory quotient (RQ), resting metabolic rate (RMR) and middle-upper arm circumference (MUAC). GWG differed between intervention groups (LED median: 12.0 kg; VLED: 5.9 kg). No differences were noted in the type of delivery, infant birth weight, composite score, prevalence of prematurity, depression, RQ, Apgar score, MUAC, or insulin use among the four groups. Regarding components of the composite score, most infants (88.4%) were appropriate-for-gestational age (AGA) and born at a gestational age of 37–42 weeks (95.3%). With respect to the mothers, 9.3% experienced complications at delivery, with the majority being allocated at the VLED + exercise arm (*p* < 0.03). The composite score was low (range 0–2.5) for all mother-infant pairs, indicating a “risk-free” pregnancy outcome. The results indicate that adherence to a LED or VLED induces similar maternal, infant and obstetrics outcomes.

## 1. Introduction

Gestational diabetes mellitus (GDM) is a medical condition associated with short- and long-term complications resulting in adverse health outcomes for the mother and the offspring [1,2,3,4,5,6,7]. Although for a minority of cases with GDM the need for insulin is indisputable, medical nutrition therapy (MNT) constitutes an essential therapeutic option for GDM and its comorbidities, either alone or as a complementary regime [8,9,10]. Several MNT components for GDM have been set based on the results of clinical trials and systematic reviews [11,12], including the preference over foods with a low-glycemic index [13] paired with a concomitant high fiber intake [14], or the possible improved outcomes following supplementation with probiotics [15]. Due to the limited available data on the optimal energy deficit required to reduce the detrimental effects of GDM and halt excessive gestational weight gain (GWG), the ideal energy intake for women with GDM remains an unsolved issue.

Although weight loss is unanimously contraindicated during pregnancy [8,16,17,18,19,20,21,22,23,24,25], energy restriction appears to be a valid recommendation for high-risk women with GDM, including those with greater adiposity [8,12,24,26]; to date, clinical trials have offered as low as 1200 kcal daily [27,28], raising concerns over possible ketogenetic effects of very low energy diets (VLED) [29,30]. Research is still inconclusive regarding the optimal energy restriction in women with GDM. According to a recent evidence synthesis [31], women with GDM on low-energy diets achieve an improved glycemic control and overall pregnancy outcomes (except for neonatal hypoglycemia) than women adhering to the standard diet. Although LED appears to be effective [8,28,31,32,33], the exact amount of energy deficit required to achieve improved outcomes is yet unknown.

The gestational diabetes Mellitus Energy Restriction (TIMER) was a non-randomized, parallel and open-label feasibility study comparing two different energy restrictive regimes with or without personalized exercise sessions in women with GDM and evaluating their effects on the GWG, pregnancy outcomes and infant characteristics.

## 2. Materials and Methods

### 2.1. Recruitment, Allocation and Blinding

**Figure 1:**
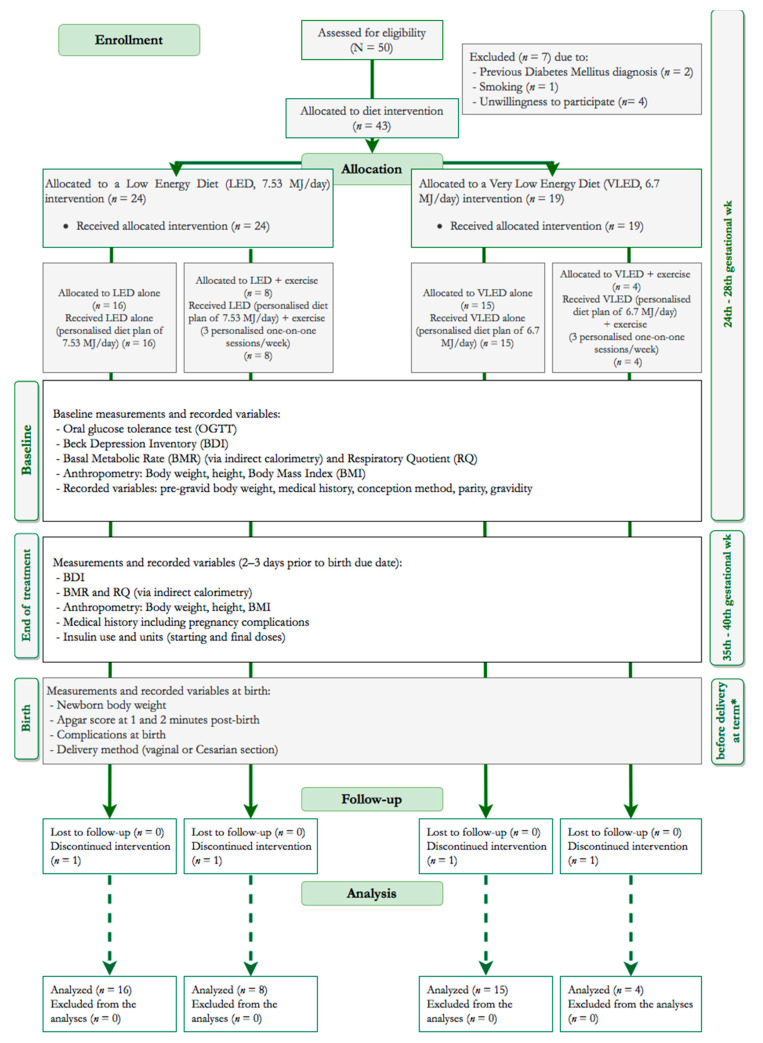
TREND diagram of the study’s process. * 2–3 days before the birth due date or imputed to the population mean when data were not available.

Singleton pregnant women with GDM were recruited from the outpatient Pregnancy Metabolic Complications clinic of a tertiary hospital (referral center) between 2014 and 2016. The recruitment of each case took place immediately after GDM diagnosis, which was based on the International Association of Diabetes and Pregnancy Study Group (IADPSG) criteria [34], following a 75 g oral glucose tolerance tests (OGTTs) between the 24th and 28th week of gestation. The participants were allocated in two groups, receiving either a low-energy diet (LED) of 1800 kcal/day, or a very low-energy diet (VLED) of 1600 kcal/day, on a 1:1 ratio. Those with greater pre-gravid BMI and middle-upper arm circumference (MUAC) were allocated to the VLED arm, as clinical practice guidelines unanimously suggest lower energy intakes for women with GDM and increased adiposity [8]. Following allocation, participants were asked to decide if they wished to receive personalized exercise programs (three sessions/week) or simple advice on increasing physical activity (PA), both lasting until delivery. Thus, the number of intervention subgroups totaled four (Figure 1). All measurements were performed at baseline (24th–28th gestational week) and at the end of the intervention (37th–38th gestational week), which is approximately 3–4 days before delivery at term date. For women who gave birth prematurely, missing values were imputed.

The participants were aware of their energy intake level and the existence of exercise groups of different intensity. Physicians, dietitians and statisticians involved in the trial were all aware of the group allocation.

### 2.2. Inclusion and Exclusion Criteria

Participants were included in the study provided that (1) they had been diagnosed with GDM according to the IADPSG criteria [34]; (2) they were adults; (3) in a singleton pregnancy; (4) in a gestational week not exceeding the 28th; (5) with a pre-gestational body mass index (BMI) between 25 and 40 kg/m^2^ or a pre-gestational BMI denoting normal body weight but with other risk factors associated with increased pregnancy-related complications, including a history of pregnancy complications, positive family history of diabetes mellitus, in-vitro fertilization (IVF) [35,36], etc.; (6) with a low frequency of rigorous physical activity involvement during the past months (<2 times per week); and (7) willing to participate.

Exclusion criteria involved pregnant women (1) with pre-existing diabetes mellitus (type 1 or 2); (2) adolescents; (3) smokers or frequent alcohol consumers; (4) with coexisting thyroid disease, chronic kidney/liver disease, malignancies or mental health diagnoses; (5) receiving corticosteroids, progesterone or chemotherapeutic drugs; and (6) unwilling to participate.

Additional exclusion criteria for women allocated in the personalized PA program were (1) history of pregnancy loss (anytime during their previous pregnancies); (2) vaginal hemorrhage; (3) placental abnormalities; or (4) reported early contractions.

### 2.3. Dietary Intervention Characteristics

In all four intervention groups, participants were prescribed a personalized diet plan of either 6.7 MJ (1600 kcal) (VLED) or 7.52 MJ (1800 kcal) (LED) daily. All women received advice and nutrition education sessions by a registered dietitian-nutritionist (RDN) (Ef.T.) before participation and throughout gestation, which were on-demand and on a 24/7 basis, as MNT delivered by RDNs has been shown to improve pregnancy outcomes [37]. Following group allocation, nutrition education sessions were performed at each visit to the clinic and through telephone calls while focusing on consuming low glycemic index foods, limiting sugar and fat consumption, increasing vegetable intake and becoming savvy concerning the diabetes exchange system [8,38].

All groups received a total of 175 g of carbohydrates daily, mainly of low glycemic index, equally divided between the three main meals (breakfast, lunch and dinner) and three snacks (brunch, afternoon and before sleep snack) [16,19,21,23,26,39,40,41]. A total of 28 g of dietary fibers were provided each day and protein intake was calculated individually, based on the Academy of Nutrition and Dietetics recommendations (1.1 g/kg of body weight/day) [26].

### 2.4. Exercise and Physical Activity Advice Interventions

In each calorie intake group, participating women were asked to choose between two subgroups of exercise interventions: Those receiving advice on maintaining adequate PA levels based on the existing guidelines [42] through walking (walking group) and those participating in one-on-one personalized exercise sessions with a physical education specialist (D.S.) (exercise group).

Personalized exercise sessions were supervised by a specialized trainer (D.S.). They consisted of three sessions per week, each of 50–60 min duration, on 60–70% of the maximum heart rate (HR_max_) and/or based on a scale of 13–14 according to the Borg rating scale of perceived exertion [43]. During the first weekly session, aerobic exercise was performed, the second session focused on muscle strengthening techniques and the third weekly session combined both aerobics and muscle strengthening exercises. On the days with an exercise session, participants consumed an additional 200 kcal irrespective of group allocation (LED or VLED). Women in the LED/VLED plus exercise arms followed the same exercise regime. The duration of the exercise intervention ranged between 8–10 weeks, depending on the timing of GDM diagnosis and the initiation of the intervention for each patient.

Women allocated to receiving standard advice on maintaining adequate PA (walking subgroups) were asked to walk three times per week for 60 min, based on the existing guidelines [42].

### 2.5. Anthropometrics and Energy Requirements

The resting metabolic rate (RMR) of participants was measured at baseline and 37th–38th week of gestation using indirect calorimetry and the O_2_ dilution method, with an accuracy of 0.1% (TrueOne 2400, Parvo Medics, Salt Lake City, UT, USA). In parallel, the respiratory quotient (RQ) was calculated by dividing the amount of CO_2_ in L by the volume of consumed O_2_ in L.

The participants’ body weight at the beginning of pregnancy was reported. Both weight and stature were measured at baseline and the end of the intervention by an experienced dietitian (Ef.T.) using a Seca 700 mechanical scale with an attached stadiometer (Seca, Hamburg, Germany). Women were advised to wear light clothing and all measurements were performed in the morning hours. The BMI was calculated for each participant by dividing the body mass (kg) by the height (m) squared.

For each participant, MUAC was measured at baseline and the end of intervention with a common anelastic tape for the non-dominant arm (left according to most participants) as a measure of the nutritional status of the participating women. Clinical practice guidelines suggest that those with a MUAC exceeding 33 cm are over-nourished, whereas women with a MUAC < 23 cm suffer from malnutrition [21].

### 2.6. Blood Glucose and Ketone Levels Measurements

The participants were advised to self-monitor blood glucose concentrations each day, both at fasting and postprandial state [8,26,39].

Urine ketone concentrations were also measured twice daily (morning, night) with urine sticks. When blood glucose concentrations exceeded 240 mg/dL, the participants were advised to check their urine for ketones. The same procedure was repeated several times per day when participants felt unwell. In parallel, blood ketone concentrations were measured [8,26] three times throughout the intervention; in cases exceeding 1.5 mmol/L, an additional 100 kcal was added to the daily dietary intake.

### 2.7. Dietary Treatment Adherence

Adherence to the dietary regime was evaluated by an experienced registered dietitian (Ef.T.) during the study visits and through frequent telephone calls and the use of 24 h dietary recalls. Participants described their intake regarding the previous day in detail, including the quantity and quality of the consumed food. Adherence to the energy intake was assessed by calculating the total energy intake (TEI) of previous day recalls, using the food equivalents and comparing TEI to the level of prescribed energy. The 24 h recall method is a retrospective short-term method for assessing dietary and food intake, exhibiting high validity and reproducibility when administered by experts [44].

### 2.8. Maternal Characteristics and Additional Measurements

Recorded maternal characteristics involved conception method, gravidity and parity. Preeclampsia was diagnosed in those with gestational hypertension (blood pressure ≥ 140/90 mm Hg on at least two measurements that were separated by a 4 h interval, after the 20th week of gestation) and coexisting proteinuria (protein urine concentrations ≥ 300 mg/24 h or positive results on a dipstick test on at least two repetitions and separated by a 6 h interval) [45]. The psychological status of the participants was evaluated with the Beck depression inventory-II (BDI-II) [46], which has been previously translated and validated in the Greek language [47].

### 2.9. Fetal Outcomes

Mode of delivery (cesarean section/vaginal birth), gestational age and complications at birth were recorded for all. Prematurity was defined as birth before the 37th week of gestation [48,49]. Birth weight was measured by an experienced midwife (El.T.) immediately after birth. Infants with <2.5 kg of weight at birth were considered low-birth-weight and those with a weight > 4.0 kg were diagnosed with macrosomia [50]. The INTERGROWTH-21st Consortium birth weight standards [51] were used to diagnose large-for-gestational-age (LGA) infants as those with a weight exceeding the 90th percentile based on their gestational age. According to the same reference curves, small-for-gestational-age (SGA) infants were diagnosed with lower birth weight than the 10th percentile [51]. Those with a birth weight between the 10th and 90th percentile for their gestational age were considered as appropriate-for-gestational age (AGA) [51]. In premature infants, the Fenton [52] growth charts were used to diagnose LGA, AGA and SGA infants accordingly.

The Apgar scoring [53] was used to assess possible signs of hemodynamic compromise and resuscitation response if required. It was recorded at 1 min and 5 min post-birth for all infants. Apgar scores at 5 min post-birth were considered optimal when ranging between 7–10, suboptimal when ranging between 4–6 and low in cases when scores < 4 were recorded [54].

### 2.10. Composite Outcome Score

A composite outcome score, which was adapted by previous research [55,56], was calculated for each mother-infant pair based on the prevalence of stillbirth, pregnancy duration, birth weight, preeclampsia and cesarean section delivery. Every mother–infant pair received a score based on the existence and severity of the five outcomes mentioned above, as suggested by Shen [56]. The score ranges from 0 (ideal pregnancy and delivery) to 100, with greater scores indicating worse outcomes.

### 2.11. Primary Outcome Measures

Primary outcome measures were total GWG, infant birth weight (kg), complications at delivery and the composite outcome score.

### 2.12. Secondary Outcome Measures

Secondary outcomes included type of delivery (vaginal/cesarean), prematurity, birth of SGA/LGA infants, birth of low birth weight (LBW) or macrosomic infants, Apgar score at 1 min and 5 min post-birth, insulin use, initiation week and final insulin units (pre-delivery), as well as change (Δ) in the BDI, RQ, RMR and MUAC post-intervention.

### 2.13. Adverse Events

In case of adverse events, the participants could call the RD-endocrinology resident in charge (Ef.T.) and receive advice concerning any issue. Possible ketonuria incidents were recorded and an experienced dietitian (Ef.T.) intervened via telephone and provided advice on consuming more energy via rapidly absorbed carbohydrates.

### 2.14. Ethical Approval and Informed Consent

Ethical approval for the study was granted by the Medical School, Aristotle University of Thessaloniki (A7922/18-04-2011).

Each participant was informed of the nature of the study and provided informed consent before participation. The protocol for the present study was registered at the Open Science Framework (OSF, https://bit.ly/3w2ihjr, accessed on 17 July 2021). Each participant had the option to resign from the study at any time and for any reason.

### 2.15. Missing Data and Analysis Plan

A modified intention-to-treat (ITT) analysis was performed according to Polit and Gillespie [57]. When data were missing, the mean value of the sample was used to replace them (imputation to the mean) [58].

### 2.16. Statistical Analyses

Normality was tested using the Shapiro–Wilk test. Normally distributed variables were presented as mean ± standardized deviation (SD), whereas non-normally distributed variables were presented as median with the respective interquartile range (IQR). In the four group analyses, group differences were evaluated using non-parametric tests (namely the Kruskal–Wallis test) as some groups demonstrated very few entries (≤4). Categorical variables were examined either with Fisher’s exact test or the chi-squared test. Statistical significance was set at 0.05. Analyses were conducted using the Jamovi Package (version 0.9.5.16, The Jamovi Project) [59]. The statistical significance was set at alpha ≤ 0.05. When *p* was equal to 0.05, the confidence intervals were additionally used.

Although the present study was designed using four intervention groups, during the review process it was decided that presenting the results comparing the two dietary intervention groups (LED vs. VLED) only, would additionally benefit future research. Subsequently, these findings were also included in the present analyses. Differences in continuous variables were analyzed using the *t*-test (for normally distributed variables) or the Mann–Whitney U test (for non-normally distributed variables). Qualitative variables were analyzed using the Fisher’s exact test or the chi-squared test.

## 3. Results

### 3.1. Baseline Characteristics and Drop-Outs

The baseline characteristics of participants of all four groups are presented in Table 1. One patient in each subgroup discontinued the intervention due to scheduled conflict with the study visits and for finding it difficult to adhere to treatment (total *n* = 4); however, they were included in the analyses (ITT).

At conception, 13 women (33.3%) were obese, 28.2% (*n* = 11) were overweight and the remaining were of normal body weight. At the beginning of the intervention, six women had a MUAC indicative of being over-nourished (≥33 cm). Overall, women in the VLED arm had a greater mean MUAC at baseline compared to the women allocated to the LED intervention.

When the two dietary intervention arms were compared at baseline, MUAC was significantly greater in the VLED compared to the LED intervention, as was expected (Table 1).

### 3.2. Maternal and Fetal Outcomes

Table 2 details the maternal and infant outcomes, delivery particularities, as well as components of the composite outcome score and the total score for each mother-infant pair. Total GWG differed between intervention arms, with participants in the LED subgroup gaining the most weight (median of 12.0 kg) and those in the VLED arm receiving a median of 6.0 kg until delivery. No differences were noted in the use of insulin (initiation time or units) among participating groups. Out of 43 women with GDM, approximately half (*n* = 22, 51.2%) initiated insulin therapy.

Regarding infant birth weight, infants demonstrated a similar weight at delivery regardless of the maternal intervention. Type of delivery (vaginal or C-section) and prevalence of prematurity were also indifferent between groups, with only two infants being born immature and both originating from the VLED subgroups. None of the participating women gave birth after the 39th week of gestation.

No cases of macrosomia were recorded in the sample. Most infants (88.4%) were classified as AGA, 4.7% were SGA and 7% were LGA, without any differences between intervention subgroups. Infant sex and Apgar scores were also similar between interventions. Approximately 9.3% (*n* = 4) of the participating mothers experienced complications at delivery, with the majority (*n* = 2) being allocated at the VLED + exercise intervention, although this finding was not significant.

Regarding the individual components of the maternal-fetal outcomes score, no cases of stillbirth or preeclampsia were noted (Table 2). C-section was employed for the delivery of 55.8% of the cases. The majority of infants (95.3%) were born at a gestational length of 37–42th weeks, with only two being born at a gestational age of 34–36 weeks, both stemming from the VLED and VLED + exercise arms (one from each subgroup). No differences were noted regarding gestational week at delivery, birth weight or composite maternal-fetal outcomes score between groups. For all participating mother–infant pairs, the composite score was extremely low and ranged between 0 and 2.5, which is indicative of a “risk-free” pregnancy outcome.

In Table 3, maternal and infant outcomes, delivery particularities and components of the composite outcome score are presented regarding the two dietary intervention arms only (LED and VLED). Total GWG appeared lower in the VLED arm, although not statistically significant. No differences were noted between the two groups.

Table 4 describes the changes in selected outcomes in each group, from the beginning of the intervention until delivery between the four intervention arms. Throughout pregnancy, the RMR of participants was similarly increased in all four interventions, with the greatest rise being recorded among women in the exercise subgroups.

No differences were recorded in the Δ RQ between intervention arms, which reveals the adoption of a similar diet composition among participants. In parallel, none of the participants exhibited an RQ below 0.7, which indicates underfeeding or the use of ketone as the main energy fuel [60].

The MUAC difference from baseline was similar between groups at the end of the intervention. At birth, seven women exhibited a MUAC exceeding 33 cm, which is indicative of over-nutrition, belonging to the LED (*n* = 2) and VLED (*n* = 5) treatment arms.

With regard to depression, no differences were noted between the four intervention arms.

Table 5 presents the recorded changes in selected outcomes from baseline to the end of intervention and between the two dietary intervention arms (LED vs. VLED). No differences were recorded in the Δ in RMR, RQ, MUAC or BDI score between the two interventions.

### 3.3. Adverse Events

No adverse events were reported in either intervention arm. Three women from the VLED group experienced mild, moderate and severe urine ketones during the intervention (two on the first and one on the last week of intervention) than compared to two cases of mild ketone levels in the LED arm (both on the first week of intervention) (Table 6). To correct this phenomenon, an additional 100 kcal/day was added to their daily energy intake. Throughout the intervention, no differences were recorded in the prevalence of positive urine ketones between intervention arms on each consecutive week.

When positive urine ketone levels between the two dietary intervention groups were compared, no differences were noted between the LED and VLED arms (*p* = 0.59).

## 4. Discussion

The present non-randomized clinical study provided no evidence of differences in maternal-birth outcomes among pregnant women diagnosed with GDM following a LED or VLED, with or without a parallel personalized exercise intervention. Moreover, regardless of the intervention arm, no difference was noted in the composite outcomes score between groups and this indicates that all four interventions induced a similar minimal risk.

MNT is an important part of GDM, with RCTs suggesting that the delivery of MNT by RDNs reduces the number of women in need of insulin therapy and improves clinical and medical outcomes [61,62]. Moreover, lifestyle interventions for GDM, including diet and exercise, have been shown to reduce inflammation markers during the third trimester of pregnancy [63]. In parallel, it appears that the physiological effects of lifestyle interventions exert beyond the delivery date [64]. According to Wexler [65], research gaps in GDM are evident and involve, in particular, carefully designed energy-balance studies. Subsequently, prescribing the ideal energy intake for women with GDM remains a challenge, with VLED being prescribed for women with severe obesity and multiple comorbidities. Currently, most clinical practice guidelines for the management of GDM suggest heterogeneous recommendations regarding energy restriction [8]. Moreover, according to Reader [66], caloric restriction is often suggested in the outpatient setting, without, however, defining specific energy goals. The present study failed to reveal differences in the outcomes produced by adherence to a VLED or a LED, suggesting that the application of more restrictive diets may be omitted and a LED may be prescribed to all women with GDM to ensure improved adequacy in the nutrient intake and to promote euglycemia and adequate weight gain. In line with the present findings, Rae [33] failed to record differences in the adverse effects of energy restriction among pregnant women with GDM using an RCT design, which provided 1572 or 1635 kcal daily, until delivery. At the moment, we are awaiting the results of more trials, with the DiGest (Dietary intervention in Gestational diabetes) RCT allocating women with GDM to 2000 or 1200 kcal/day [67].

In the present study, details concerning the macronutrient content of the diet were not provided to the participants herein, with the exception of carbohydrate intake goals. Most clinical practice guidelines for the management of GDM advocate for incorporating carbohydrate counting in the educational sessions provided to future mothers [8]. More recent recommendations on the optimal GDM diet suggest restricting carbohydrate intake instead of energy [39,68]. In a small RCT [69], women allocated to the modestly lower carbohydrate (MLC) diet had concomitantly reduced their energy intake (mean intake 1682 kcal/day) compared with the routine care group participants; however, no differences in the GWG or birth outcomes were noted between intervention and comparator arms. Similar results were also reported in a previous RCT [70].

No differences were recorded in the number of LGA and SGA infants born between intervention arms, which indicates that the degree of energy restriction and the incorporation of exercise did not induce different infant growth and classification outcomes at birth. A previous study conducted in Greece revealed a higher prevalence of LGA and SGA among women with GDM, indicating that the intervention might have tampered down the prevalence herein [71]. Snyder [72] was the first to identify the importance of pre-gravid BMI in the infant birth weight when GDM is diagnosed. In this case, lifestyle interventions should precede conception. According to a later study [73], maternal GWG consists of an additional strong infant birth weight effector. On this basis, it is possible that the energy restriction applied in all intervention arms herein produced a similar effect and did not allow for differences in the prevalence of SGA or LGA infants between intervention groups.

Moreover, in the VLED arms, two cases of prematurity were noted, although the incidence was not significantly different compared with the LED interventions. Due to the small number of cases and the lack of any family data indicating a high prematurity risk in these women, it is not safe to make any conclusions on this particular issue.

The RQ is a useful tool in planning diet therapy. Normal RQ values range from 0.7 to 1.2, depending on the relative contribution of macronutrients to the diet intake [60]. In adults, increased RQs are associated with increased body weight and fat mass gain [74]. During gestation, a rise in RQ occurs during the third trimester [75,76,77] due to adaptations in carbohydrate and fat metabolism, which marks the shift from an anabolic to a catabolic state [78]. In the present study, the four distinct interventions failed to induce differences in the RQ between groups, which indicates that participants followed a diet of similar content.

According to the results, MUAC did not differ between the treatment arms at the end of the intervention. MUAC is considered a better surrogate index of body fat in pregnancy compared with the BMI [79]. In parallel, MUAC is strongly correlated with the prevalence of GDM [80,81]. Unfortunately, due to the small number of participants, we could not perform further analyses concerning this anthropometric index.

Research appears unanimous concerning the increased efficacy of combined diet and exercise interventions for the treatment of GDM and for improving pregnancy outcomes [3,42,82,83]. Nevertheless, it appears that a GDM diagnosis alone does not motivate women to increase their PA levels [84,85]. In GDM, apart from reducing GWG, exercise is also tightly related to the glycemic control, with lower PA levels being associated with increased blood glucose concentrations [86,87]. In parallel, exercise can also reduce the need for insulin [88] by facilitating glucose uptake in the skeletal muscle. According to Chatzakis [89], exercise has several physiological effects, including the promotion of additional glucose uptake through improved translocation of the insulin-responsive glucose transporters (GLUT4) on the cell surface and the enhanced glucose skeletal muscle uptake in the presence of insulin [89]. These two pathways induce an improved insulin sensitivity and a reduced GWG [89]. Combined interventions with energy restriction and exercise can also mediate obesity-induced inflammation [88]. Overall, women’s awareness regarding the optimal mode, intensity and duration of PA sessions during gestation appears to be suboptimal [90]. In the present study, very few participants opted for the combined diet and exercise interventions despite being supervised. Indeed, most of the studies on women with GDM have applied supervised exercise interventions and, according to Onaade, this information is difficult to translate into everyday clinical practice [87].

In the present study, the recorded incidence of C-sections was high compared to other studies. In particular, Greece is known to have a higher rate of performed C-sections [91], ranging from a mean of 41.6% recorded rate in the public setting to 53% in the private health sector [92]. Therefore, the recorded high rate herein does not appear to be the epiphenomenon of VLED or LED but is instead a reflection of a common practice among obstetricians throughout the country.

Not all GDM cases can be considered the same. The differential impact of lifestyle interventions on GDM highlights the need for tailored patient interventions instead of horizontal, generalized recommendations [65]. According to Reader [66], maternal adaptations to pregnancy influencing caloric requirements include pre-gravid nutritional status, a rise in RMR and a reduction in PA levels. All these factors should be accounted for when recommending nutrient targets and assessing diet goals for women with GDM.

Limitations of the present study include the small number of participants, as this was a pilot feasibility study. Moreover, the lack of randomization might increase selection bias and influence the findings. As for the lack of objective adherence assessment methods, such as doubly labeled water, this might have resulted in reduced adherence to the prescribed interventions. Nevertheless, due to the lack of studies prescribing reduced energy intake during pregnancy, the present findings are important in adding to the limited existing evidence regarding optimal energy intake in women with GDM. Moreover, the exact diet composition of participants could not be assessed as only 24 h dietary recalls were recorded, which focused mainly on the adherence to the energy restriction patterns. As a result, it is not easy to assess whether participants achieved nutrient and food group goals and whether adequate standards were retained with respect to diet quality. Concerning the use of composite outcomes scores, according to Profit [93], their use facilitates benchmarking and summarizes complex issues for guidelines authors and decision-makers; it also entails few limitations when lacking basic conceptual skills. However, in the present study, we opted for using a composite score previously applied in several research protocols, including research on GDM [94].

## 5. Conclusions

To date, the existing evidence indicate that combined diet and exercise interventions are successful in improving obstetrical, maternal and fetal outcomes in women with GDM [95] and successful for reducing the risk for intergenerational obesity [68,96,97]. The present study showed that VLED and LED therapies do not appear to induce differences in the aforementioned outcomes among women with GDM. Nevertheless, more research is required to validate the present findings and to identify the ideal energy intake goal for women with GDM.

## Figures and Tables

**Table 1:** Participant characteristics at baseline (mean ± SD, median and IQR, or *n*).

Parameters	LED (*n* = 16)	LED + Exercise (*n* = 8)	VLED (*n* = 15)	VLED + Exercise (*n* = 4)	*p* Value (4 arms)	*p* Value (LED vs. VLED)
Age (years)	34.4 ± 4.99	32.6 ± 4.24	33.9 ± 3.78	36.8 ± 2.99	0.45 ^1^	0.75 ^3^
Pre-gravid body weight (kg)	76.3 ± 17.3	70.8 ± 11.9	86.2 ± 17.0	69.0 ± 13.5	0.10 ^1^	0.11 ^3^
Height (cm)	167 (162–174)	168 (165–169)	167 (161–170)	160 (160–163)	0.48 ^1^	0.42 ^3^
Pre-gravid BMI (kg/m^2^)	25.3 (23.1–31.5)	26.3 (23.1–28.1)	32.1 (26.8–35.0)	23.8 (23.2–26.9)	0.08 ^1^	0.05 ^4^
MUAC (cm)	28.3 ± 3.92	28.7 ± 2.85	31.5 ± 3.18	28.8 ± 2.53	0.04 ^1^	0.02 ^3^
Method of conception Spontaneous/IUI/IVF (*n*)	16/0/0	6/1/1	14/1/0	4/0/0	0.23 ^2^	0.48 ^2^
Gravidity 1st/2nd/3rd/4th/5th/6th (*n*)	5/7/1/1/1/1	5/3/0/0/0/0	3/10/1/1/0/0	1/2/0/1/0/0	0.75 ^2^	0.84 ^2^
Parity 0/1/2/3 (*n*)	8/6/1/1	6/2/0/0	4/11/0/0	2/1/1/0	0.11 ^2^	0.14 ^2^
RMR (kcal/day)	1781 (1662–1851)	1689 (1581–1945)	1842 (1604–2150)	1811 (1613–1909)	0.82 ^1^	0.47 ^4^
RQ	0.790 (0.760–0.843)	0.805 (0.800–0.833)	0.810 (0.775–0.855)	0.820 (0.808–0.837)	0.66 ^1^	0.67 ^3^
BDI	10.0 (7.75–15.0)	8.50 (7.25–13.5)	10.0 (6.00–11.0)	8.00 (7.50–8.50)	0.61 ^1^	0.36 ^4^

BDI, Beck Depression Inventory; BMI, Body Mass Index; LED, Low energy diet (1800 kcal/day); IQR, interquartile range; IUI, Intrauterine insemination; IVF, in vitro fertilization; MUAC, middle-upper arm circumference; RMR, Resting Metabolic Rate; RQ, Respiratory quotient; SD, standard deviation; VLED, Very low energy diet (1600 kcal/day). ^1^ Based on the Kruskal–Wallis test due to the small number of participants in group 4. ^2^ Based on the Fisher’s exact test. ^3^ According to the Student’s *t*-test ^4^ According to the Mann–Whitney U test, due to violation of the normality assumption.

**Table 2:** Maternal, obstetrical and infant outcomes between intervention groups (presented as median and IQR or *n*, %).

		LED (*n* = 16)	LED + Exercise (*n* = 8)	VLED (*n* = 15)	VLED + Exercise (*n* = 4)	*p* Value
Total gestational weight gain (kg)		12.0 (9.8–14.3)	10.3 (8.9–11.5)	6.0 (2.6–9.3)	9.0 (6.7–12.5)	0.03 ^1^
Insulin use:	Initiation week	24 (21–28)	30 (29–30)	22 (15–28)	25 (24–25)	0.15 ^1^
	Units at labor (IU)	16 (9–33)	14 (11–21)	26 (19–30)	13 (11–16)	0.44 ^1^
	Insulin users (*n*)	8 (50.0%)	4 (50.0%)	8 (53.3%)	2 (50.0%)	1.00 ^2^
Infant birth weight (g)		3120 (2815–3283)	2980 (2948–3255)	2950 (2750–3245)	3275 (3115–3485)	0.47 ^1^
Type of delivery:	Vaginal (*n*)	8 (50.0%)	5 (62.5%)	5 (33.3%)	1 (25.0%)	0.48 ^2^
	C-section (*n*)	8 (50.0%)	3 (37.5%)	10 (66.7%)	3 (75.0%)	
Prematurity (*n*)		0 (0.0%)	0 (0.0%)	1 (6.7%)	1 (25.0%)	0.14 ^2^
Infant birth weight categorization:	AGA (*n*)	13 (81.3%)	8 (100.0%)	14 (93.3%)	3 (75.0%)	0.52 ^2^
	SGA (*n*)	1 (6.3%)	0 (0.0%)	1 (6.7%)	0 (0.0%)	
	LGA (*n*)	2 (12.5%)	0 (0.0%)	0 (0.0%)	1 (25.0%)	
Infant sex:	Female/Male (*n*, %)	10/6 (62.5%/37.5%)	4/4 (50.0%/50.0%)	8/7 (53.3%/46.7%)	3/1 (75.0%/25.0%)	0.86 ^2^
Apgar score:	at 1 min post-birth	8.0 (8.0–8.0)	9.0 (8.0–9.0)	8.0 (8.0–8.0)	8.0 (8.0–8.0)	0.12 ^1^
	at 5 min post-birth	9.0 (9.0–9.0)	9.0 (9.0–9.0)	9.0 (9.0–9.0)	9.0 (9.0–9.0)	0.23 ^1^
Complications at delivery (*n*)		1 (6.3%)	0 (0%)	1 (6.7%)	2 (50.0%)	0.07 ^2^
Composite outcome score components:	Stillbirth (*n*)	0	0	0	0	-
	Preeclampsia (*n*)	0	0	0	0	-
	C-section delivery (*n*)	8 (50.0%)	3 (37.5%)	10 (66.7%)	3 (75.0%)	0.48 ^2^
	Gestational age 24–34 wk (*n*)	0	0	0	0	
	Gestational age 34–36 wk (*n*)	0	0	1 (6.7%)	1 (25.0%)	0.14 ^2^
	Gestational age ≥ 37 wk, <42 wk (*n*)	16 (100%)	8 (100%)	14 (93.3%)	3 (75.0%)	
	Gestational age > 42 wk (*n*)	0	0	0	0	
	Birth weight < 750 g (*n*)	0	0	0	0	
	Birth weight 750–1500 g (*n*)	0	0	0	0	
	Birth weight 1500–2500 g (*n*)	1 (6.3%)	0	1 (6.7%)	0	1.00 ^2^
	Birth weight 2500–4000 g (*n*)	15 (93.8%)	8 (100%)	14 (93.3%)	4 (100%)	
	Birth weight > 4000 g (*n*)	0	0	0	0	
Composite maternal-fetal score		1.3 (0.0–2.5)	0.0 (0.0–2.5)	2.5 (0.0–2.5)	2.5 (2.4–2.5)	0.36 ^1^

AGA, appropriate-for-gestational age; C-section, cesarean section; IQR, interquartile range; IU, International Units; LED, low energy diet (1800 kcal/day); LGA, large-for-gestational age; SGA, small-for-gestational age; VLED, very low energy diet (1600 kcal/day). ^1^ According to the Kruskal–Wallis test. ^2^ Based on the Fisher’s exact test.

**Table 3:** Maternal, obstetrical and infant outcomes between the two dietary intervention arms (presented as median and IQR or *n*, %).

Parameters		LED (*n* = 16)	VLED (*n* = 15)	*p* Value
Total gestational weight gain (kg)		11.4 ± 5.0	6.8 ± 7.6	0.05 ^1^
Insulin use	Initiation week	25 ± 7	22 ± 8	0.36 ^1^
	Units at labor (IU)	18.9 ± 13.7	26.8 ± 13.6	0.27 ^1^
	Insulin users (*n*)	8 (50.0%)	8 (53.3%)	0.85 ^2^
Infant birth weight (g)		3114 ± 400	3011 ± 333	0.44 ^1^
Type of delivery	Vaginal (*n*)	8 (50.0%)	5 (33.3%)	0.35 ^2^
	C-section (*n*)	8 (50.0%)	10 (66.7%)	
Prematurity (*n*)		0 (0.0%)	1 (6.7%)	0.48 ^3^
Infant birth weight categorization	AGA (*n*)	13 (81.3%)	14 (93.3%)	0.73 ^3^
	SGA (*n*)	1 (6.3%)	1 (6.7%)	
	LGA (*n*)	2 (12.5%)	0 (0.0%)	
Infant sex: Female/Male (*n*)		10/6 (62.5%/37.5%)	8/7 (53.3%/46.7%)	0.61 ^2^
Apgar score:	at 1 min post-birth	8.0 (8.0–8.0)	8.0 (8.0–8.0)	0.55 ^4^
	at 5 min post-birth	9.0 (9.0–9.0)	9.0 (9.0–9.0)	1.00 ^4^
Complications at delivery (*n*)		1 (6.3%)	1 (6.7%)	1.00 ^3^
Composite outcome score components	Stillbirth (*n*)	0	0	
	Preeclampsia (*n*)	0	0	
	C-section delivery (*n*)	8 (50.0%)	10 (66.7%)	0.35 ^2^
	Gestational age 24–34 wk (*n*)	0	0	
	Gestational age 34–36 wk (*n*)	0	1 (6.7%)	
	Gestational age ≥ 37 wk, <42 wk (*n*)	16 (100%)	14 (93.3%)	0.48 ^3^
	Gestational age > 42 wk (*n*)	0	0	
	Birth weight < 750 g (*n*)	0	0	
	Birth weight 750–1500 g (*n*)	0	0	
	Birth weight 1500–2500 g (*n*)	1 (6.3%)	1 (6.7%)	
	Birth weight 2500–4000 g (*n*)	15 (93.8%)	14 (93.3%)	1.00 ^3^
	Birth weight > 4000 g (*n*)	0	0	
	Composite maternal-fetal score	1.3 (0.0–2.5)	2.5 (0.0–2.5)	0.41 ^4^

AGA, appropriate-for-gestational age; C-section, cesarean section; IQR, interquartile range; IU, International Units; LED, low energy diet (1800 kcal/day); LGA, large-for-gestational age; SGA, small-for-gestational age; VLED, very low energy diet (1600 kcal/day). ^1^ According to Student’s *t*-test. ^2^ Based on the chi-squared test. ^3^ Based on the Fisher’s exact test. ^4^ According to the Mann–Whitney U test, due to violation of the normality assumption.

**Table 4:** Change (Δ) in the selected outcomes from the beginning to the end of the intervention in each group (median and IQR).

	LED (*n* = 16)	LED + Exercise (*n* = 8)	VLED (*n* = 15)	VLED + Exercise (*n* = 4)	*p* Value ^1^
RMR (kcal/day)	177.0 (37.3 to 265.0)	279.0 (184.0 to 318.0)	121.0 (−51.0, to 339.0)	231.0 (147.0 to 360.0)	0.56
RQ	0.025 (−0.003 to 0.060)	0.00 (−0.013 to 0.03)	0.010 (−0.005 to 0.050)	−0.025 (−0.053 to −0.018)	0.12
MUAC (cm)	1.0 (−0.50 to 1.50)	−0.10 (−1.00 to 0.50)	−1.20 (−1.50 to 0.150)	−0.85 (−1.50 to −0.15)	0.11
BDI score	0.00 (−1.00 to 2.00)	−0.50 (−1.00 to 2.00)	0.00 (−1.00 to 1.00)	1.00 (0.50 to 2.50)	0.85

BDI, Beck Depression Inventory; IQR, interquartile range; LED, low energy diet (1800 kcal/day); MUAC, middle-upper arm circumference; RMR, Resting Metabolic Rate; RQ, Respiratory Quotient; VLED, very low energy diet (1600 kcal/day). ^1^ According to the Kruskal–Wallis test.

**Table 5:** Change (Δ) in the selected outcomes from the beginning to the end of the intervention in each dietary intervention arm (median and IQR).

	LED (*n* = 16)	VLED (*n* = 15)	*p* Value ^1^
RMR (kcal/day)	170.0 (37.3 to 265.0)	121.0 (−58.0 to 339.0)	0.92
RQ	0.025 (−0.003 to 0.060)	0.010 (−0.005 to 0.050)	0.61
MUAC (cm)	1.00 (−0.50 to 1.50)	−1.20 (−1.50 to 0.35)	0.07
BDI score	0.00 (−1.00 to 2.00)	0.00 (−1.00 to 1.00)	0.75

BDI, Beck Depression Inventory; IQR, interquartile range; LED, low energy diet (1800 kcal/day); MUAC, middle-upper arm circumference; RMR, Resting Metabolic Rate; RQ, Respiratory Quotient; VLED, very low energy diet (1600 kcal/day). ^1^ According to the Mann–Whitney U test and due to violation of the normality assumption.

**Table 6:** Cumulative urine ketone results based on the dipstick test during the total intervention duration, in each arm.

Ketone Urine Value	LED (*n* = 16)		LED + Exercise (*n* = 8)		VLED (*n* = 15)		VLED + Exercise (*n* = 4)		Total	
	*n*	*%*	*n*	*%*	*n*	*%*	*n*	*%*	*n*	*%*
0 (negative)	142	98.60%	72	100%	132	97.80%	36	100%	382	98.70%
+1 (mild)	2	1.40%	0	0%	1	0.70%	0	0%	3	0.80%
+2 (moderate)	0	0%	0	0%	1	0.70%	0	0%	1	0.26%
+3 (ketonuria)	0	0%	0	0%	1	0.70%	0	0%	1	0.26%
Total	144		72		135		36		387	

LED, low energy diet (1800 kcal/day); VLED, very low energy diet (1600 kcal/day).

## Data Availability

Data are available upon request from the senior author (D.G.G.).
